# Variations in the Binding Pocket of an Inhibitor of the Bacterial Division Protein FtsZ across Genotypes and Species

**DOI:** 10.1371/journal.pcbi.1004117

**Published:** 2015-03-26

**Authors:** Amanda Miguel, Jen Hsin, Tianyun Liu, Grace Tang, Russ B. Altman, Kerwyn Casey Huang

**Affiliations:** 1 Department of Bioengineering, Stanford University, Stanford, California, United States of America; 2 Department of Microbiology and Immunology, Stanford University School of Medicine, Stanford, California, United States of America; Heidelberg Institute for Theoretical Studies (HITS gGmbH), GERMANY

## Abstract

The recent increase in antibiotic resistance in pathogenic bacteria calls for new approaches to drug-target selection and drug development. Targeting the mechanisms of action of proteins involved in bacterial cell division bypasses problems associated with increasingly ineffective variants of older antibiotics; to this end, the essential bacterial cytoskeletal protein FtsZ is a promising target. Recent work on its allosteric inhibitor, PC190723, revealed *in vitro* activity on *Staphylococcus aureus* FtsZ and *in vivo* antimicrobial activities. However, the mechanism of drug action and its effect on FtsZ in other bacterial species are unclear. Here, we examine the structural environment of the PC190723 binding pocket using PocketFEATURE, a statistical method that scores the similarity between pairs of small-molecule binding sites based on 3D structure information about the local microenvironment, and molecular dynamics (MD) simulations. We observed that species and nucleotide-binding state have significant impacts on the structural properties of the binding site, with substantially disparate microenvironments for bacterial species not from the *Staphylococcus* genus. Based on PocketFEATURE analysis of MD simulations of *S*. *aureus* FtsZ bound to GTP or with mutations that are known to confer PC190723 resistance, we predict that PC190723 strongly prefers to bind *Staphylococcus* FtsZ in the nucleotide-bound state. Furthermore, MD simulations of an FtsZ dimer indicated that polymerization may enhance PC190723 binding. Taken together, our results demonstrate that a drug-binding pocket can vary significantly across species, genetic perturbations, and in different polymerization states, yielding important information for the further development of FtsZ inhibitors.

## Author Summary

The rise of antibiotic resistance in microbes that cause dangerous diseases necessitates the development of new drugs with novel mechanisms of antimicrobial activity. The recently identified small molecule PC190723 is a promising therapeutic candidate, as it is the only drug known to directly inhibit FtsZ, a cytoskeletal protein that is critical for bacterial cell division. However, little is known about the effects of PC190723 in bacterial species other than the pathogen *Staphylococcus aureus*. Here, we evaluate the PC190723 binding site with currently available crystallographic structures of FtsZ using the structural comparison algorithm PocketFEATURE and all-atom molecular dynamics simulations. Our investigations provide insight into the mechanism of PC190723-based inhibition of FtsZ and its potential use as an inhibitor of as-yet untested bacterial species. We also demonstrate the utility of computational analysis of the drug-binding pockets of related proteins, which we expect to accelerate the screening and development of novel antibiotics to combat human disease.

## Introduction

Rises in bacterial antibiotic resistance have motivated the development of new classes of drugs with alternative mechanisms of action [1]. One promising target is the cytoskeletal protein FtsZ, a GTPase and homolog of eukaryotic tubulin that plays a central, conserved role in cell division in both eubacteria and archaea [2]. FtsZ forms protofilaments *in vitro* [3] and assembles *in vivo* into a ring-like structure termed the “Z-ring,” which acts as a scaffold to recruit other critical division proteins [4] and constricts the cell as division progresses [5,6]. *In vitro* studies have shown that FtsZ causes constriction when bound to liposomes, either via a membrane-targeting helix or with its *in vivo* binding partner FtsA, suggesting that FtsZ can generate mechanical force in addition to its scaffolding function [7–9]. All-atom molecular dynamics (MD) simulations predicted that force generation may result from a dramatic bending in GDP-bound filaments induced by nucleotide hydrolysis [10]; this conformational change was later confirmed by X-ray crystallography [6].

**Fig 1:**
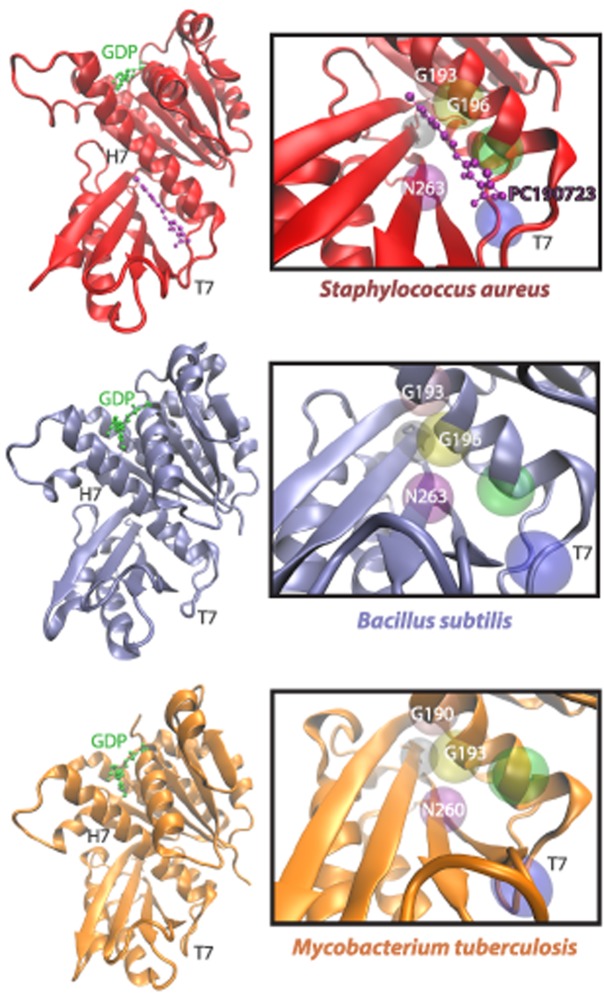
The FtsZ PC190723-binding pocket differs structurally across bacterial species. PC190723 binds a cleft beneath the H7 helix and adjacent to the T7 loop of FtsZ, as demonstrated in an *Sa*FtsZ-PC197023 co-crystal (PDB ID: 4DXD) [17], in a *B*. *subtilis* FtsZ (PDB ID: 2RHL) structure [42], and in a *Mycobacterium tuberculosis* FtsZ (PDB ID: 1RQ7) structure [43]. Highlighted above are a few of the 20 amino acid residues that are within 6 Å of PC190723, including G193, G196, and N263, which induce drug resistance when mutated in *Sa*FtsZ.

Several compounds have been demonstrated to inhibit FtsZ, primarily through three mechanisms [11]: modulation of FtsZ assembly/disassembly [12,13], GTPase activity [14,15], or degradation [16]. However, many of these compounds have as-yet unknown interactions with FtsZ, large minimal inhibitory concentrations, or are associated with toxic effects that make them unsuitable for therapeutic use [11]. In comparison, the FtsZ inhibitor PC190723 and its derivatives are promising drug candidates. PC190723 inhibited cell division at low minimal inhibitory concentration in many Gram-positive bacteria, such as the model organism *Bacillus subtilis* and the pathogen *Staphylococcus aureus*, including the methicillin-resistant *S*. *aureus* strain [12]. Encouragingly, PC190723 exhibited antimicrobial properties *in vivo* in mouse models [12], suggesting that this small molecule selectively targets bacterial cell division without affecting tubulin, the eukaryotic FtsZ homolog. Furthermore, single point mutations conferring resistance to PC190723 in *S*. *aureus* were identified within *ftsZ*, suggesting that PC190723 specifically binds to *S*. *aureus* FtsZ (*Sa*FtsZ). This observation was later confirmed via the co-crystallization of an *Sa*FtsZ-PC190723 complex in two independent studies [17,18]; thus, currently PC190723 is the only FtsZ-targeting drug with evidence of direct binding. In both co-crystals, PC190723 binds a pocket beneath the H7 loop close to the C-terminus of FtsZ, and many of the FtsZ residues with identified resistance mutations (for example, G193, G196, and N263) lie within 6 Å of the PC190723 molecule in these structures (Fig. 1). Although the PC190723-binding pocket is quite far from FtsZ’s GTP-binding site, it is situated near the T7 loop, which is thought to contact the GTP-binding pocket of an adjacent FtsZ subunit after polymerization, forming the catalytic active site for GTP hydrolysis [19].

While PC190723 inhibits cell division in a variety of bacterial species such as *S*. *aureus* and *B*. *subtilis*, its exact mechanism remains unclear. The initial study of PC190723 showed that addition of PC190723 reduced *Sa*FtsZ GTPase activity *in vitro* [12]; in contrast, a recent study reported that PC190723 actually increased *Sa*FtsZ GTPase activity, and exhibited no effect on the GTPase activity of *B*. *subtilis* FtsZ (*Bs*FtsZ) [20,21]. *In vitro*, PC190723 induced stable bundling of *Sa*FtsZ and *Bs*FtsZ filaments; for FtsZ from *Escherichia coli*, a Gram-negative bacterium whose growth is not inhibited by PC190723 treatment, no such bundling was observed [22]. The diverse behaviors observed after PC190723 treatment in FtsZ from diverse species potentially indicate structural complexity in the drug-binding site that has yet to be explored.

Here, we present a structural analysis of the PC190723-binding pocket of FtsZ from multiple species and across a variety of conformational states. We characterized this binding pocket using PocketFEATURE, a statistical algorithm that performs pairwise comparisons of structural information between small molecule-binding pockets in different proteins [23]. This method was previously used to predict inhibitor binding profiles between kinases [23]. PocketFEATURE divides a ligand-binding pocket into a set of microenvironments based on local structural and chemical information, and scores the similarity between this set of microenvironments with those found in other proteins. Using PocketFEATURE, we compared the PC190723-binding pocket from the *Sa*FtsZ-PC190723 co-crystal with FtsZ crystal structures from multiple bacterial and archaeal species, including the Gram-positive *S*. *aureus*, *B*. *subtilis*, and *Mycobacterium tuberculosis*, the Gram-negative *Pseudomonas aeruginosa*, and the archaeon *Methanocaldococcus jannaschii* (Table 1). The resulting PocketFEATURE score is a measure of the similarity of that pocket to that of the *Sa*FtsZ-PC190723 co-crystal. PocketFEATURE scores revealed that the microenvironment of the PC190723-binding pocket is dependent on species and the nucleotide-binding state of the protein, with the best similarity scores belonging to *Staphylococcus* species bound to a GDP nucleotide. Similarity scores computed from the coordinates of all-atom MD simulations preserved the ranking order determined by their static crystal structures counterparts, with PC190723-resistant *Sa*FtsZ mutants harboring pockets that were less similar to the *Sa*FtsZ-PC190723 co-crystal than wild-type *Sa*FtsZ pockets. Furthermore, all FtsZ proteins, except those from *Staphylococcus* species, had pockets that were no more similar to the *Sa*FtsZ-PC190723 co-crystal structure than PC190723-resistant *Sa*FtsZ mutants. Finally, FtsZ polymerization increased the pocket similarity of wild-type *Sa*FtsZ to the *Sa*FtsZ-PC190723 co-crystal, but not that of a PC190723-resistant mutant. Taken together, our results suggest that PC190723 binds best to polymerized, nucleotide-bound FtsZ of *Staphylococcus* species.

**Table 1:** Description of FtsZ crystal structures for which PocketFEATURE scores were computed.

Organism	PDB	Ligand(s)	Chain	Pocket score	Resolution (Å)
*Aquifex aeolicus*	2R6R	GDP	1	-4.124	1.7
*Aquifex aeolicus*	2R75	8-morpholino-GTP, magnesium	1	-3.755	1.4
*Bacillus subtilis*	2RHH	sulfate	A	-6.813	2
*Bacillus subtilis*	2RHJ	sodium, Glycol, sulfate, acetate	A	-6.837	1.76
*Bacillus subtilis*	2RHL	GDP	A	-7.161	2.45
*Bacillus subtilis*	2RHL	GDP	B	-7.124	2.45
*Bacillus subtilis*	2RHO	GDP, GTPγS	A	-6.96	2.45
*Bacillus subtilis*	2RHO	GDP, GTPγS	B	-6.722	2.45
*Bacillus subtilis*	2VAM	sulfate	A	-7.413	2.5
*Bacillus subtilis*	2VXY	citrate, potassium	A	-6.673	1.7
*Methanocaldococcus jannaschii*	2VAP	GDP	A	-4.264	1.7
*Methanocaldococcus jannaschii*	1W58	G2P (ester), magnesium	A	-3.29	2.5
*Methanocaldococcus jannaschii*	1W59	sulfate	A	-2.932	2.7
*Methanocaldococcus jannaschii*	1W59	sulfate	B	-2.825	2.7
*Methanocaldococcus jannaschii*	1W5A	GTP, magnesium	A	-3.642	2.4
*Methanocaldococcus jannaschii*	1W5A	GTP, magnesium	B	-3.147	2.4
*Methanocaldococcus jannaschii*	1W5B	GTP	A	-3.324	2.2
*Methanocaldococcus jannaschii*	1W5B	GTP	B	-3.582	2.2
*Methanocaldococcus jannaschii*	1FSZ	GDP	A	-2.621	2.8
*Mycobacterium tuberculosis*	1RLU	GTPγS, glycerol	A	-5.899	2.08
*Mycobacterium tuberculosis*	1RLU	GTPγS, glycerol	B	-7.024	2.08
*Mycobacterium tuberculosis*	1RQ2	citrate	A	-7.146	1.86
*Mycobacterium tuberculosis*	1RQ2	citrate	B	-7.051	1.86
*Mycobacterium tuberculosis*	2Q1X	citrate	A	-6.672	2.35
*Mycobacterium tuberculosis*	2Q1X	citrate	B	-6.098	2.35
*Mycobacterium tuberculosis*	1RQ7	GDP	A	-6.867	2.6
*Mycobacterium tuberculosis*	1RQ7	GDP	B	-7.362	2.6
*Mycobacterium tuberculosis*	2Q1Y	GTPγS	A	-6.889	2.3
*Mycobacterium tuberculosis*	2Q1Y	GTPγS	B	-7.099	2.3
*Mycobacterium tuberculosis*	4KWE	GDP	A	-2.916	2.91
*Mycobacterium tuberculosis*	4KWE	GDP	B	-3.656	2.91
*Mycobacterium tuberculosis*	4KWE	GDP	C	-2.919	2.91
*Pseudomonas aeruginosa*	1OFU	GDP	A	-4.052	2.1
*Pseudomonas aeruginosa*	1OFU	GDP	B	-4.154	2.1
*Pseudomonas aeruginosa*	2VAW	GDP	A	-4.945	2.9
*Staphylococcus aureus*	3VO8	GDP, Calcium	A	-10.82	2.26
*Staphylococcus aureus*	3VO8	GDP, Calcium	B	-10.754	2.26
*Staphylococcus aureus*	3VO9	Selenomethionine	A	-5.784	2.71
*Staphylococcus aureus*	3VO9	Selenomethionine	B	-4.298	2.71
*Staphylococcus aureus*	3VO9	Selenomethionine	C	-4.243	2.71
*Staphylococcus aureus*	3VO9	Selenomethionine	D	-5.201	2.71
*Staphylococcus aureus*	3VOA	GDP, Calcium	A	-10.755	1.73
*Staphylococcus aureus*	3VOB	GDP, 9PC	A	-11.201	2.7
*Staphylococcus aureus*	3VPA	APO	A	-4.32	2.49
*Staphylococcus aureus*	3VPA	APO	B	-4.917	2.49
*Staphylococcus aureus*	3VPA	APO	C	-4.909	2.49
*Staphylococcus aureus*	3VPA	APO	D	-5.109	2.49
*Staphylococcus aureus*	4DXD	GDP, 9PC	1	-12	2.01
*Staphylococcus epidermidis*	4M8I	GDP	A	-10.001	1.43
*Thermobifida fusca*	4E6E	chloride, magnesium, sulfate	A	-3.471	2.12

## Results

### PC197023 pocket scores from FtsZ crystal structures are highly species-dependent

**Fig 2:**
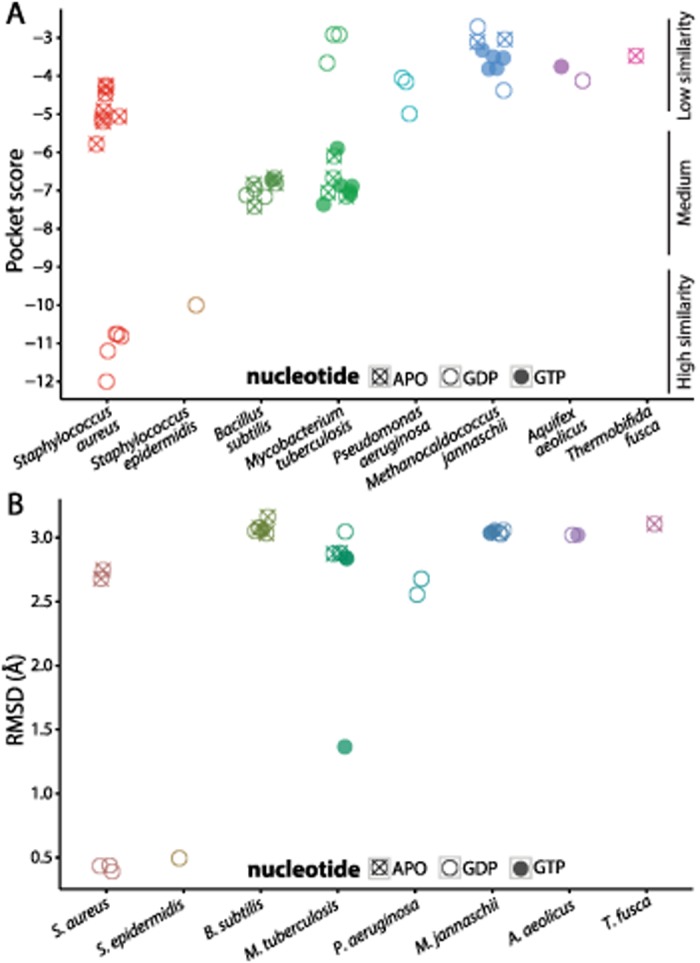
PC197023-binding pocket similarity scores depend on species and nucleotide-binding state. (A) PocketFEATURE scores for FtsZ structures from various species compared to the PC190723-binding pocket of *Sa*FtsZ (PDB ID: 4DXD). GDP-bound *Staphylococcus* species display more similarity (more negative scores) to the *Sa*FtsZ-PC190723 co-crystal, with APO *Staphylococcus* structures and structures from other bacterial species showing less similarity to the *Sa*FtsZ-PC190723 co-crystal. (B) RMSD of the 3D coordinates of FtsZ crystal structures from the PC190723 co-crystal coarsely separate the proteins into close and distant relations of GDP-bound *Staphylococcus* FtsZ, but this measurement does not capture additional features of the drug pockets that distinguish between structures of medium and low similarity to the *Sa*FtsZ-PC190723 co-crystal.

Crystal structures of *Sa*FtsZ bound to PC190723 (PDB ID: 4DXD and 3VOB) [17,18] suggest that PC190723 induces antimicrobial activity in this organism by specifically binding FtsZ, but no evidence of direct binding exists in other bacterial species. To probe how species-specific features of the PC190723-binding pocket’s three-dimensional microenvironment affect the drug’s affinity for FtsZ, we mapped the PC190723-binding pocket onto FtsZ molecules from various species (Fig. 1, S1 Fig, Methods) and computed scores for their similarity to the *Sa*FtsZ-PC190723 co-crystal (Fig. 2A, Table 1). The similarity scores roughly fell into categories of high, medium, and low similarity. GDP-bound structures of *Sa*FtsZ and *Staphylococcus epidermidis* FtsZ (*Se*FtsZ) were highly similar to the binding pocket in the *Sa*FtsZ-PC190723 co-crystal, consistent with a previous report that both species are sensitive to PC190723 [12]. Interestingly, crystal structures of *Sa*FtsZ had similar pocket scores to each other whether or not PC190723 was bound (Fig. 2A). In contrast, we obtained worse similarity scores for FtsZ from all other species; *B*. *subtilis* (*Bs*FtsZ) and *M*. *tuberculosis* (*Mt*FtsZ) structures had only intermediate similarity to the *Sa*FtsZ-PC190723 co-crystal, despite evidence of PC190723 sensitivity in *B*. *subtilis* [12]. FtsZ structures from all other species were associated with comparatively poor similarity scores (Fig. 2A), suggesting that these pockets are less conducive to PC190723 binding. Notably, binding site microenvironment comparison performed by PocketFEATURE captures details that are not detected solely through structural analysis. Root mean squared deviation (RMSD) of the pockets of the crystal structures to the pocket of the *Sa*FtsZ-PC190723 co-crystal (Fig. 2B) clearly identified that *Staphylococcus* species have pocket structures that are more similar to that of the *Sa*FtsZ-PC190723 co-crystal, but in contrast to PocketFEATURE, RMSD is unable to distinguish among non-*Staphylococcus* species.

### PC197023 pocket score is dependent on nucleotide state

For *Sa*FtsZ, we also noted a marked decrease in similarity score for the APO state (no nucleotide) relative to GDP-bound structures, with APO scores similar to that of *Bs*FtsZ (Fig. 2A). This observation suggests that conformational changes associated with nucleotide binding may tune the binding affinity of PC190723 for FtsZ; no GTP-bound *Sa*FtsZ structures exist, leaving it unclear whether GTP hydrolysis significantly affects pocket score. Interestingly, there did not appear to be any nucleotide dependence in *Bs*FtsZ (Fig. 2A), and *Mt*FtsZ displayed the opposite tendency to *Sa*FtsZ (Fig. 2A), with worse similarity to the *Sa*FtsZ-PC190723 co-crystal for GDP-bound structures relative to GTP-bound or APO structures. However, all *Bs*FtsZ and *Mt*FtsZ binding pockets have medium to low similarity compared to GDP-*Sa*FtsZ, and thus may not be optimal for PC190723 binding regardless of nucleotide state. These data suggest that the identity of the nucleotide bound to an FtsZ monomer has variable effects on PC190723 binding across species. In the case of *Sa*FtsZ, pocket scores predict that PC190723 is more likely to bind FtsZ when GDP is also bound to it.

### Resistance mutations substantially reduce PC190723 pocket scores

**Fig 3:**
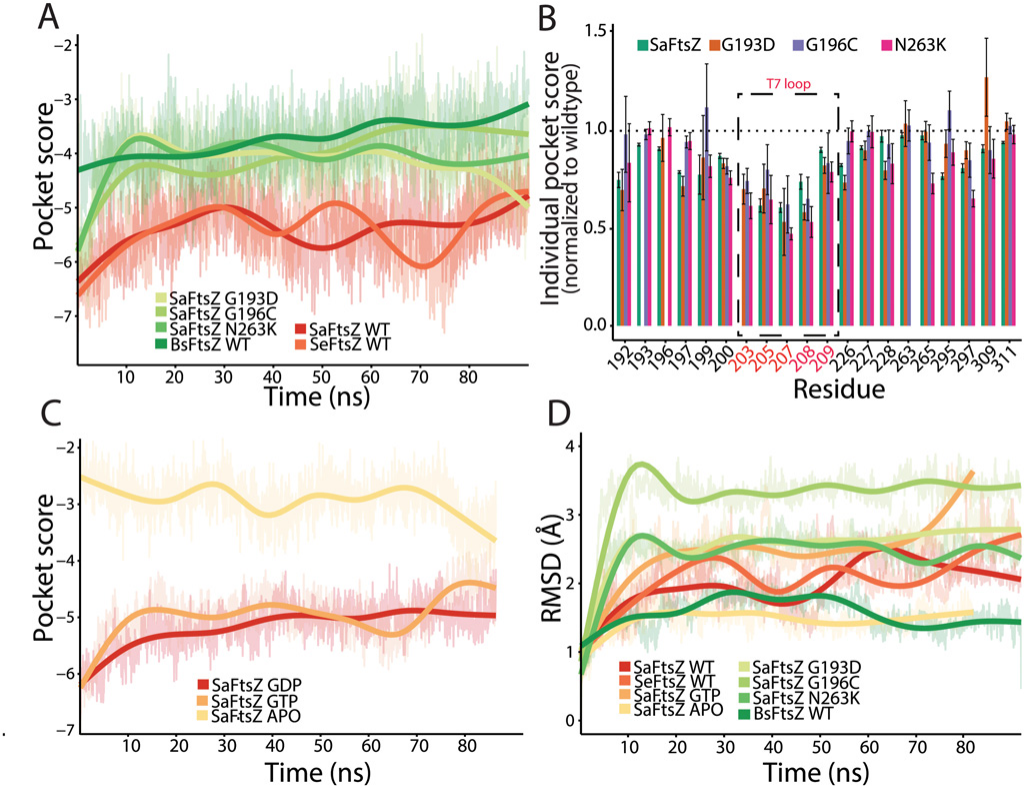
MD simulations predict that PC190723 binding affinity is reduced in GTP-bound *Sa*FtsZ and PC190723-resistant mutants compared with GDP-bound *Sa*FtsZ. (A) PocketFEATURE scores of dynamic FtsZ monomer structures distinguish among the FtsZ structures that bind PC190723 (*S*. *aureus* and *S*. *epidermidis*), PC190723-resistant *S*. *aureus* mutants (G193D, G196C, N263K), and *Bs*FtsZ. WT, wildtype. (B) Individual residue contributions to the PocketFEATURE scores show that certain residues in the T7 loop (203, 205, 207) contribute less to the overall score in PC190723-resistant mutants compared to wildtype. (C) Pocket scores from MD trajectories depend on nucleotide, with GDP- and GTP-bound FtsZ exhibiting significantly better similarity to the co-crystal compared to the APO state. (D) RMSD of pocket residues over simulation trajectories do not capture the same hierarchy of pocket similarity scores as in (A,C). Thick lines in (A,C,D) represent a smoothed version of the raw data (Methods).

We sought to determine if FtsZ mutations distort the microenvironment of the PC190723 binding site. We therefore compared the pockets of wild-type *Sa*FtsZ with those of the PC190723-resistant mutants G193D, G196C, and N263K [12]. Since crystal structures of these mutants do not exist, we conducted all-atom MD simulations on *Sa*FtsZ GDP-bound wildtype and drug-resistant (mutant) monomers in order to evaluate how the mutations affected the PC190723-binding pocket. All *Sa*FtsZ simulations were initialized from the crystal structure of the GDP-bound *Sa*FtsZ without PC190723 (PDB ID: 3VO8) (Fig. 3A, Methods). We observed an initial, rapid decline in similarity to the static FtsZ-PC190723 co-crystal for all simulated monomers. Close investigation into the individual microenvironments contributing to the pocket similarity score for each frame revealed that while the majority of the 20 residues included in the pocket score computation remained within the binding pocket, some had decreased contribution to the total similarity score. For example, over the simulation trajectory of the wild-type *Sa*FtsZ monomer, an average of 17 out of the 20 residues contributed to the similarity score at any given time point during the simulation, resulting in an overall shift in the PocketFEATURE score to less negative values (decreased similarity). Thus, small fluctuations in the amino acid functional centers within the pocket can perturb the local physiochemical properties calculated for each residue, pointing to the subtle structural balance required for the most optimal drug-binding environment that can be extremely sensitive to thermal fluctuations. Nevertheless, the time-scale and magnitude of this change as the simulations reached a steady state were consistent across all FtsZ monomers (Fig. 3A), indicating that comparisons between simulated protein structures can be established.

To compare pocket scores from MD trajectories across species, we also carried out MD simulations of *Se*FtsZ and *Bs*FtsZ monomers initialized from their crystal structures (PDB IDs: 2RHL and 4M8I, respectively); we observed similar initial decreases in pocket similarity (Fig. 3A). Despite the general change in pocket score to the *Sa*FtsZ-PC190723 co-crystal in all simulations relative to the pocket score computed from static crystal structures, *Sa*FtsZ and *Se*FtsZ monomers maintained better similarity to the *Sa*FtsZ-PC190723 co-crystal compared to the *Bs*FtsZ monomer. These results are consistent with the comparisons of pocket score magnitudes determined from static crystal structures (Fig. 2A), indicating that important features of the PC190723-binding pocket are conserved in MD simulations.

All three *Sa*FtsZ mutant monomers had significantly worse pocket scores in comparisons with the *Sa*FtsZ-PC190723 co-crystal than a wild-type monomer, and similar scores to that of *Bs*FtsZ. Further examination of the microenvironment pocket scores of the *Sa*FtsZ mutants and *Bs*FtsZ (Fig. 3B) revealed that a subset of residues (203–207 in *S*. *aureus*) across all four simulations had average score magnitudes below those of wild-type *Sa*FtsZ. Interestingly, these residues correspond to amino acids within the T7 loop, a highly conserved structural element near the site of monomer association that plays an important role in GTP hydrolysis [19]. In *E*. *coli*, mutations affecting GTP hydrolysis occur either in the nucleotide pocket or in the T7 loop [2,19]. Truncations of this loop in *Sa*FtsZ disrupt hydrolysis [24]. Taken together, affinity of PC190723 to FtsZ is likely closely coupled to the FtsZ structural transition that occurs during the hydrolysis cycle, and mutations in FtsZ can sufficiently disrupt the PC190723 binding environment.

### Absence of nucleotide, but not nucleotide hydrolysis, strongly affects PC190723 pocket score

Given the large difference in pocket scores in comparisons with the *Sa*FtsZ-PC190723 co-crystal between APO and GDP-bound *Sa*FtsZ crystal structures, we wondered whether it was nucleotide binding or hydrolysis that had a greater effect on pocket microenvironment. Since there are no GTP-bound crystal structures of *Sa*FtsZ, we conducted MD simulations of the APO state and GTP-bound *Sa*FtsZ monomers (Fig. 3C) to complement our GDP-bound monomer simulations (Fig. 3A). Due to the potential for small atomic differences between the GTP- and GDP-bound states of FtsZ, we conducted three independent simulations of each monomer (Fig. 3C). For each replicate, we observed that GTP- and GDP-bound *Sa*FtsZ had similar drug-pocket scores, and better similarity scores than the APO state. These results illustrate the sensitivity of the drug pocket to allosteric effects of the nucleotide bound on the opposite side of the protein, and suggest that occupancy of the nucleotide-binding site, rather than the identity of the nucleotide located there, alters the conformation of the pocket to favor PC190723 binding.

To test whether the chemical and physical properties evaluated by PocketFEATURE are captured by traditional structural-only metrics such as RMSD, we calculated the RMSD of the 20 pocket residues aligned to the same 20 residues of the 4DXD *Sa*FtsZ-PC190723 co-crystal structure for each MD trajectory (Fig. 3D). Large-magnitude pocket scores did not correspond with low RMSD from the *Sa*FtsZ-PC190723 co-crystal, with *Sa*FtsZ and *Se*FtsZ having values intermediate between the low values of APO *Sa*FtsZ and *Bs*FtsZ and the high values of *Sa*FtsZ^G193D^. Taken together, these data indicate that PocketFEATURE provides a distinctly informative metric for comparing similarity between pockets, and that PC190723 binding may be unique to *Staphylococcus* species.

### FtsZ polymerization improves the PC190723 pocket score

**Fig 4:**
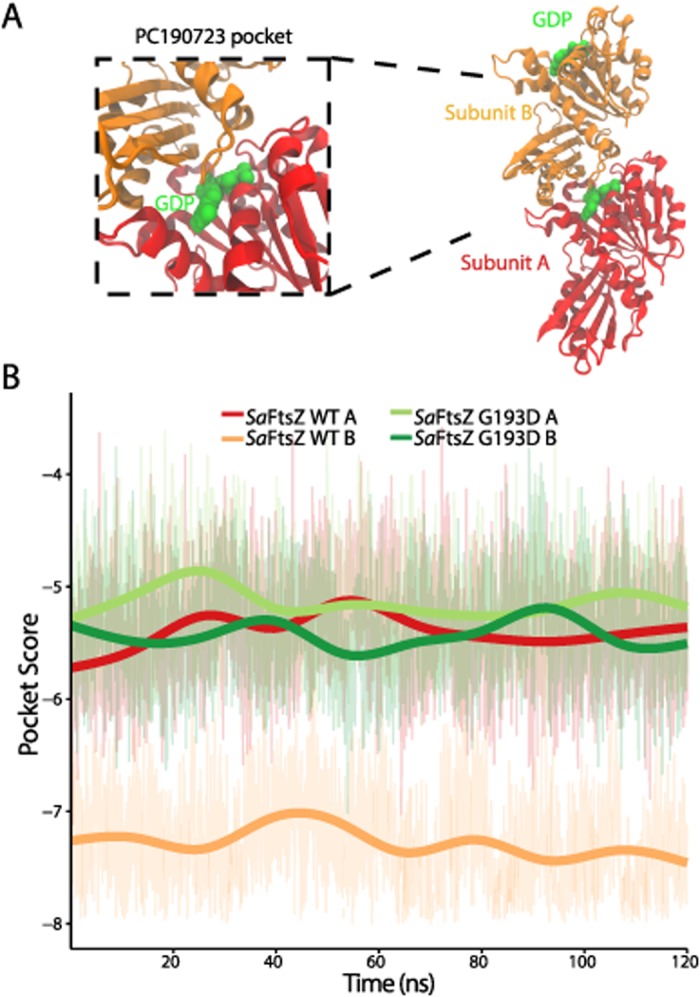
Pocket similarity score predicts that polymerization increases PC190723 binding affinity. (A) Geometry of the FtsZ dimer structure. FtsZ subunits were initialized from the GDP-bound *S*. *aureus* wild-type structure (PDB ID: 3VO8). (B) PocketFEATURE scores of the dynamic FtsZ dimer structure show that the PC190723-binding pocket close to the dimer interface has significantly more pocket similarity to the *Sa*FtsZ-PC190723 co-crystal than the more exposed pocket on the end of the dimer. Thick lines are smoothed versions of the raw data (Methods). WT, wildtype.

FtsZ hydrolysis and polymerization are intrinsically coupled. Key catalytic residues for GTP hydrolysis reside on the T7 loop of the adjacent subunit near the dimer interface [19], and mutations near the active site designed to modulate hydrolysis also affect the dynamics of polymer assembly [25]. Since the PC190723-binding pocket is located close to this interface, we hypothesized that it may be affected by FtsZ polymerization. We conducted MD simulations of GDP-bound dimers of wild-type *Sa*FtsZ (Fig. 4A) and the G193D mutant, and examined the pocket scores of each subunit of the dimers (Fig. 4B). In the subunits of the wild-type dimer, we observed a statistically significant difference (t-test, *p* < 2.2e-16) in pocket scores, with the subunit with the pocket closest to the dimer interface having a better similarity score to the *Sa*FtsZ-PC190723 co-crystal (Fig. 4B). No such difference was evident in the PC190723-resistant G193D mutant dimer; both subunits had pocket scores throughout the trajectory that were similar to those of a wild-type subunit with a pocket away from the dimer interface (Fig. 4B).

At later times in the MD simulation, the PC190723 pocket score of the wild-type dimer subunit with the better pocket score rapidly shifted to a value similar to that of the other subunit with poorer similarity (S2A Fig). To probe the cause of this change, we examined the conformations of the wild-type dimer from 130–180 ns. We observed a conformational shift at the dimer interface that opens the GDP-binding pocket, likely as a prelude to the release of the bound nucleotide (S2B Fig). Given that this biologically relevant release of GDP at the dimer interface has a corresponding drastic affect on the PC190723-binding pocket in our MD simulations (S2A Fig), this further confirms the allosteric coupling between the bound nucleotide and PC190723 binding. Since we were interested in the PC190723-binding pocket in a stable GDP-bound dimer, we focused on the pocket similarity score during the first 120 ns of the trajectory (Fig. 4B), which showed that polymerization increases the pocket similarity to the *Sa*FtsZ-PC190723 co-crystal as compared with FtsZ monomers. While these calculations were made over a shorter timescale than that required for the system to reach convergence, our results suggest that polymerization induces pocket deformations with an increased likeness to the co-crystal drug pocket.

## Discussion

Our analyses of FtsZ crystal structures and MD trajectories indicate that multiple factors including species, allosteric binding, genetic perturbations, and polymerization state all contribute to the conformations of the PC190723-binding pocket. In particular, polymerization and allosteric binding of a guanosine nucleotide play a pivotal role in stabilizing the PC190723 pocket; the drug pocket is also subject to species-level differences, despite the close structural similarities of FtsZ from all characterized species at the level of entire monomers [26]. Using all-atom MD simulations to quantify perturbations to the PC190723-binding pocket due to resistance-inducing mutations, we have provided an indirect assessment of PC190723 binding affinity that can be used as a point of comparison for at least two relevant applications. First, our results impart insight into the interpretation of experimental results suggesting different responses of *Sa*FtsZ and *Bs*FtsZ to PC190723 [20]. Second, our analyses illustrate how evaluation of the 3D environment of a drug-binding pocket can illuminate the molecular properties and mechanisms governing drug-protein interactions.

Our prediction that PC190723 has high affinity for its binding pocket in FtsZ structures from the *Staphylococcus* species but not in FtsZ from other species has important implications for its development as a future therapeutic. Previously, experimental evidence of PC190723’s antimicrobial properties in *S*. *aureus* and *B*. *subtilis* seemed to agree well with *in vitro* studies indicating that the drug acts through excess polymer stabilization [12,22]; in contrast, another *in vitro* study reported increased enzymatic activity in *Sa*FtsZ and no change in *Bs*FtsZ [20]. Our analysis indicates that the PC190723-binding pocket in *Bs*FtsZ is less similar to the *Sa*FtsZ-PC190723 co-crystal than the non-drug bound *Sa*FtsZ structure, both as a static crystal structure (Fig. 2A) and in our MD trajectories (Fig. 3A). This observation, combined with evidence from our MD simulations that *Bs*FtsZ pocket scores are on par with the scores of point mutations that confer PC190723 resistance in *S*. *aureus* (Fig. 3A), argues that *Bs*FtsZ is unlikely to strongly bind PC190723 in this particular pocket, and therefore is likely inhibited by PC190723 either via binding to an alternate, unidentified pocket or through an unknown, indirect mechanism. Our analysis of the pocket’s microenvironment indicates that a major reason for the decrease in pocket similarity is the incompatibility of the residues around the catalytically important T7 loop near the polymerization interface (Fig. 3D); in the case of *Bs*FtsZ, our data are more consistent with *in vitro* evidence that *Bs*FtsZ GTPase activity is unaffected by the addition of PC190723 [20]. Taken together, the magnitude of the drop in pocket similarity for all species other than *S*. *aureus* and *S*. *epidermidis* indicates that PC190723 may be specifically effective for binding to *Staphylococcus* FtsZ proteins and very close relatives. One limitation of the PocketFEATURE algorithm is that evaluation requires some *a priori* knowledge or estimate of a potential binding site, either from a similar protein structure or through cavity detection software. Our PocketFEATURE analysis specifically evaluated one subset of microenvironments within the pocket identified in the *Sa*FtsZ-PC190723 co-crystal; it is possible that PC190723 may bind to FtsZ from other species either through an alternative pocket or through different contacts within the same pocket. Genetic studies of mutations in FtsZ from other species that provide resistance to PC190723 would clarify whether such binding pockets exist. Evaluating whether PC190723 exerts similar antimicrobial effects on the other species we investigated in this study would yield further insight into the potential efficacy of PC190723 and its derivatives as universal, cross-species bactericides. This determination is particularly important for *M*. *tuberculosis*, another virulent bacterial species that is resistant to a variety of therapeutic drugs. Although our PocketFEATURE results indicate that the 3D microenvironments of *Bs*FtsZ and *Mt*FtsZ are similar (Fig. 2A), suggesting that PC190723 does not bind to the same pocket in *Mt*FtsZ and *Sa*FtsZ, confirmation of the inhibition of cell division in *M*. *tuberculosis* would justify the investigation of PC190723 compatibility with other potential binding pockets on the surface of *Mt*FtsZ.

Our predictions that nucleotide binding and polymerization significantly enhance PC190723 binding to *Sa*FtsZ (Fig. 4B) are consistent with experimental evidence that PC190723 stabilizes polymer formation [22]. In current models of Z-ring assembly and function, FtsZ monomers in the cytosol bind GTP, polymerize into filaments either in the cytoplasm or on the membrane, hydrolyze, and then return to the cytosol to further the constriction process [27–29]. Our simulations of APO, GDP-, and GTP-bound *Sa*FtsZ monomers (Fig. 3B) and a GDP-bound *Sa*FtsZ dimer (Fig. 4B) capture many of the FtsZ structural states at various points of this cycle, and our pocket scores indicate that the optimal PC190723-binding pocket exists when FtsZ is in a nucleotide-bound, polymerized state. Our observations that the PC190723-binding pocket is disrupted as the FtsZ dimer begins to open at the nucleotide-binding site near the dimer interface (S2 Fig) motivate future simulations of FtsZ dimers bound to PC190723 to determine whether the dimer is stabilized. Additionally, further experiments exploring PC190723 binding to an *Sa*FtsZ dimer in various nucleotide states to validate the predictions of our MD simulations of GTP-bound *Sa*FtsZ may shed light onto the mechanism by which PC190723 inhibits cellular division in *S*. *aureus*.

Our study also has important implications regarding the strengths and versatility of PocketFEATURE. By evaluating the local physical and chemical properties that dictate drug binding, we can begin to build a mechanistic understanding of the general effects of protein microenvironments. Previous applications of PocketFEATURE demonstrated its ability to distinguish similar binding pockets in static, unrelated proteins [23]. However, the significance of PocketFEATURE scores for binding affinity has only been loosely correlated. Importantly, our study showcases PocketFEATURE’s ability to distinguish pockets in structures with close homology (Fig. 3A,B) that had scores within a smaller range than was previously studied [22]. Our results establish that PocketFEATURE is able to not only distinguish aspects of the drug-binding pocket in FtsZ structures from different species that are not evident from methods such as RMSD, which only considers structural information; the algorithm also can evaluate how protein pockets in molecules from resistant mutants may differ from the optimal binding structure. PocketFEATURE can therefore aid in the design of alternative mutants with equivalent structural and chemical features. Applications of PocketFEATURE are limited in part by the dependence of similarity score evaluation on the set of existing 3D structures of protein-drug co-crystals, a data set that determines the range of pocket variability. Excitingly, our work shows that relevant differences in pocket structure can be detected in dynamic trajectories (Fig. 3A, B and 4B), highlighting the utility of MD simulations for exploring protein-drug interactions, particularly in mutant proteins whose structures have yet to be solved through X-ray crystallography or nuclear magnetic resonance.

## Methods

### Microenvironment characterization and PocketFEATURE scores

To compare inhibitor-binding pockets across FtsZ homologs, we aligned the proteins (S1 Fig) to identify the pocket residues and then utilized the PocketFEATURE method [23] in which microenvironments are defined as spherical regions around the functional centers of a residue. Utilizing the FEATURE characterization system [30], we calculated 80 physiochemical properties over six concentric spherical shells within each microenvironment, with a total microenvironment radius of 7.5 Å [30]. Each microenvironment is therefore represented by a feature vector with 480 properties. PocketFEATURE evaluates pocket similarity by combinatorially comparing the microenvironments of the two pockets and calculating a Tanimoto coefficient (*T*_*c*_) for each pair, where *T*_*c*_ is the ratio of the numbers of similar properties and total unique properties. These *T*_*c*_ scores are then evaluated against a set of equivalent microenvironment pairs derived from a dataset of 1116 sites from non-redundant 3D structures in the PDB, fit to a normal distribution, and normalized for background frequency. Microenvironments are aligned to the mutual best scoring microenvironment pairs with a normalized *T*_*c*_ score <-3. For example, for two sites A and B, microenvironments A1 and B1 will only be aligned if the scores for all other combinations of microenvironment pairs are smaller in magnitude. The PocketFEATURE binding site similarity score is the sum of the best-scoring, non-redundant microenvironment pairs. Further details of the method can be found in [23].

In this study, we define the “ideal” PC190723-binding pocket as the microenvironment of the 20 amino acid residues within 6 Å of the PC190723 molecule in an *Sa*FtsZ-PC190723 co-crystal structure (PDB ID: 4DXD) [17]. For comparison with other crystal structures, we defined the PC197023-binding pocket as the equivalent amino acids after a structural alignment (Fig. 1, S1 Fig) with the flexible structure alignment method jFATCAT on RCSB.org [31]. We then used PocketFEATURE to compare the feature lists of each protein pocket to the *Sa*FtsZ-PC190723 binding pocket and output a similarity score. This method was applied to both static FtsZ crystal structures and to dynamic structures of FtsZ simulated via MD.

### Equilibrium MD simulations

MD simulations were performed on the FtsZ crystal structures of *S*. *aureus* (PDB ID: 3VO8), *S*. *epidermidis* (PDB ID: 4M8I), and *B*. *subtilis* (PDB ID: 2RHL) using NAMD [32] with the CHARMM27 force field [33,34] and CMAP corrections [35]. Simulations were initialized from crystal structures with missing residues at the N- and C-termini, but no missing internal residues. For *S*. *aureus* FtsZ, residues 12–316 of chain A were present; for *S*. *epidermidis* FtsZ, residues 11–321; for *B*. *subtilis* FtsZ, residues 12–316. All water molecules retained in the crystal structure were removed prior to simulation and the structures were then resolvated in a box of water molecules described by the TIP3P model [36] with 14 Å padding and neutralized with NaCl using the solvate and auto-ionize VMD extensions [37]. Single point mutations were introduced into the *S*. *aureus* crystal structure with the mutate residue VMD modeling extension [37]. *S*. *aureus* FtsZ dimer structures were initialized from the 3VO8 structure. All structures underwent energy minimization prior to the equilibrium simulations. Long-range electrostatic forces were evaluated via the particle-mesh Ewald summation approach with a grid spacing of <1 Å. An integration time step of 2 fs was used [38]. Bonded terms and short-range, non-bonded terms were evaluated every time step, and long-range electrostatics were evaluated every other time step. Constant temperature (*T* = 310 K) was maintained using Langevin dynamics [39], with a damping coefficient of 1 ps^-1^. A constant pressure of 1 atm was enforced using the Langevin piston algorithm [40] with a decay period of 200 fs and a time constant of 50 fs.

For all analyses of MD simulations, amino acid pocket scores were evaluated for the last 30 ns of the trajectories to ensure structural equilibration. Smoothing was carried out with the “geom_smooth” function using a generalized additive model method in the R package ggplot2 [41].

## Supporting Information

S1 Fig Sequence alignment of FtsZ proteins from eight prokaryotic species. Blue, strictly conserved amino acids; red, highly conserved amino acids (75% conservation). The secondary structure elements of *Sa*FtsZ appear above the sequence; orange and green bars denote helices and sheets, respectively.
(EPS)

S2 Fig MD simulations predict a relationship between PocketFEATURE score and the conformation of the T7 loop. (A) 180-ns MD trajectories of FtsZ wild-type dimers (red/orange) and mutant dimers (green). A and B refer to the subunits with the PC190723 pocket exposed to the solvent and situated close to the dimer interface, respectively. At ~130 ns, we observed a large decrease in the similarity of the wild-type subunit B pocket to the *Sa*FtsZ-PC190723 co-crystal. (B) This drop is concurrent with a large change in the protein structure over the next 50 ns. By 180 ns, the GDP molecule (blue) is exposed to the solvent due to a conformational change in the T7 loop.
(EPS)
